# MiR-99b-5p and miR-203a-3p Function as Tumor Suppressors by Targeting IGF-1R in Gastric Cancer

**DOI:** 10.1038/s41598-018-27583-y

**Published:** 2018-07-04

**Authors:** Zhenzhen Wang, Zhenghao Zhao, Yang Yang, Mai Luo, Min Zhang, Xiaofei Wang, Liying Liu, Ni Hou, Qingqing Guo, Tusheng Song, Bo Guo, Chen Huang

**Affiliations:** 1 Department of Cell Biology and Genetics, School of Basic Medical Sciences, Xi’an Jiaotong University Health Science Center, Xi’an, Shaanxi P. R. China; 2 The ART Center, Northwest women’s and Children’s Hospital, Xi’an, Shaanxi P. R. China; 3 Key Laboratory of Shaanxi Province for Craniofacial Precision Medicine Research, College of Stomatology, Xi’an Jiaotong University, Xi’an, Shaanxi China; 4 Key Laboratory of Environment and Genes Related to Diseases (Xi’an Jiaotong University), Ministry of Education of China, Xi’an, Shaanxi P. R. China

**Keywords:** Gastric cancer, miRNAs

## Abstract

MicroRNAs (miRNAs) have been explored in many critical cellular processes, including proliferation and apoptosis. The purpose of this study was to detect the biological function and regulation of miR-99b-5p and miR-203a-3p in gastric cancer (GC). Here, we demonstrated that miR-99b-5p/203a-3p were downregulated in both GC tissues and cell lines. MiR-99b-5p/203a-3p overexpression reduced GC cell proliferation and cell cycle progression *in vitro*. Notably, we combined bioinformatics tools with biological validation assays to demonstrate that insulin-like growth factor 1 receptor (IGF-1R) is a direct co-target and functional mediator of miR-99b-5p/203a-3p in GC cells. Mechanistically, the AKT pathway, which is downstream of IGF-1R, is essential for the functional roles of miR-99b-5p/203a-3p in GC cells. Taken together, our data revealed that IGF-1R is a direct co-target of miR-99b-5p/203a-3p, and miR-99b-5p/203a-3p may function as tumor suppressive miRNAs by negatively regulating IGF-1R expression in GC cells.

## Introduction

Gastric cancer (GC) is one of the most common malignant diseases and the second cause of cancer mortality worldwide^1,2^. Despite of the multiple advances in clinical and experimental cancer treatment, the prognosis of GC remains poor, with a terrible 5-year survival. Numerous studies have shown that the progression of GC might be a multistep process, involving the interaction between oncogenes and tumor suppressor genes^3,4^. Consequently, a better understanding of molecular mechanisms and signaling pathways is indispensable for identification of therapeutic targets for GC.

MiRNAs are a class of highly conserved (18–23 nucleotides), non-coding RNAs that modulate target genes expression through binding to 3′-untranslated regions (3′UTR) of mRNAs, which may result in mRNA degradation and translational repression^5,6^. MiRNAs play a significant role in regulation of biological processes, including cellular apoptosis, proliferation and differentiation^7–9^. Many reports have suggested that more than half of the miRNAs are located in cancer-related genomic regions and have been identified as oncogenes or tumor suppressors, suggesting that lots of miRNAs contribute to the progression of human cancers^10–12^.

MiR-99b belongs to the members of the miR-125a-let-7e cluster and has been reported to be involved in the differentiation, migration and proliferation of cancer cells. Studies have shown that miR-99b may function as modulators within a complex network of factors regulating TGF-β induced breast epithelial to mesenchymal transition^13^. Moreover, miR-99b inhibits cervical cancer cell invasion and proliferation by targeting mTOR signaling pathway^14^. MiR-203, is located on chromosome 14q32.33, which plays an important role in the regulation of human cancers. For example, miR-203a regulates proliferation and migration by targeting glycogen synthase kinase-3β in renal cell carcinoma^15^. In GC, aberrant expression of miRNAs, such as miR-338-3p and miR-145, has been shown to regulate tumor process by targeting downstream genes^16,17^. However, the role of miR-99b-5p/203a-3p and their cellular signaling pathway in GC are less reported and need further study.

Insulin-like growth factor 1 receptor (IGF-1R) is a transmembrane receptor tyrosine kinase that primarily activated by IGF1 and IGF2^18–20^. Activated IGF-IR triggers numerous downstream signaling cascades, including mitogen-activated proteins Kinase (MAPK) and phosphatidylinositol 3-kinase (PI3K)/AKT signaling pathways that regulate carcinogenic transformation and growth of cancer cells^21–23^. Previous studies have implicated that IGF-1R was related to tumor regulation in many cancers, such as pancreatic cancer^24^, breast cancer^25,26^, lung cancer^27^ and hepatocellular carcinoma^28^. While some research has been carried out on miRNAs regulating IGF-1R expression, few studies have reported the relationship between miR-99b-5p/203a-3p and IGF-1R in GC.

Here, we investigated the roles of miR-99b-5p and miR-203a-3p in GC, which might play as suppressors, and demonstrated that IGF-1R was a co-target of miR-99b-5p/203a-3p to regulate the AKT signaling pathway. Hence, our findings facilitate better understanding of the miRNAs network control mechanism in the progression of GC.

## Results

### MiR-99b-5p and miR-203a-3p are downregulated in GC

**Figure 1:**
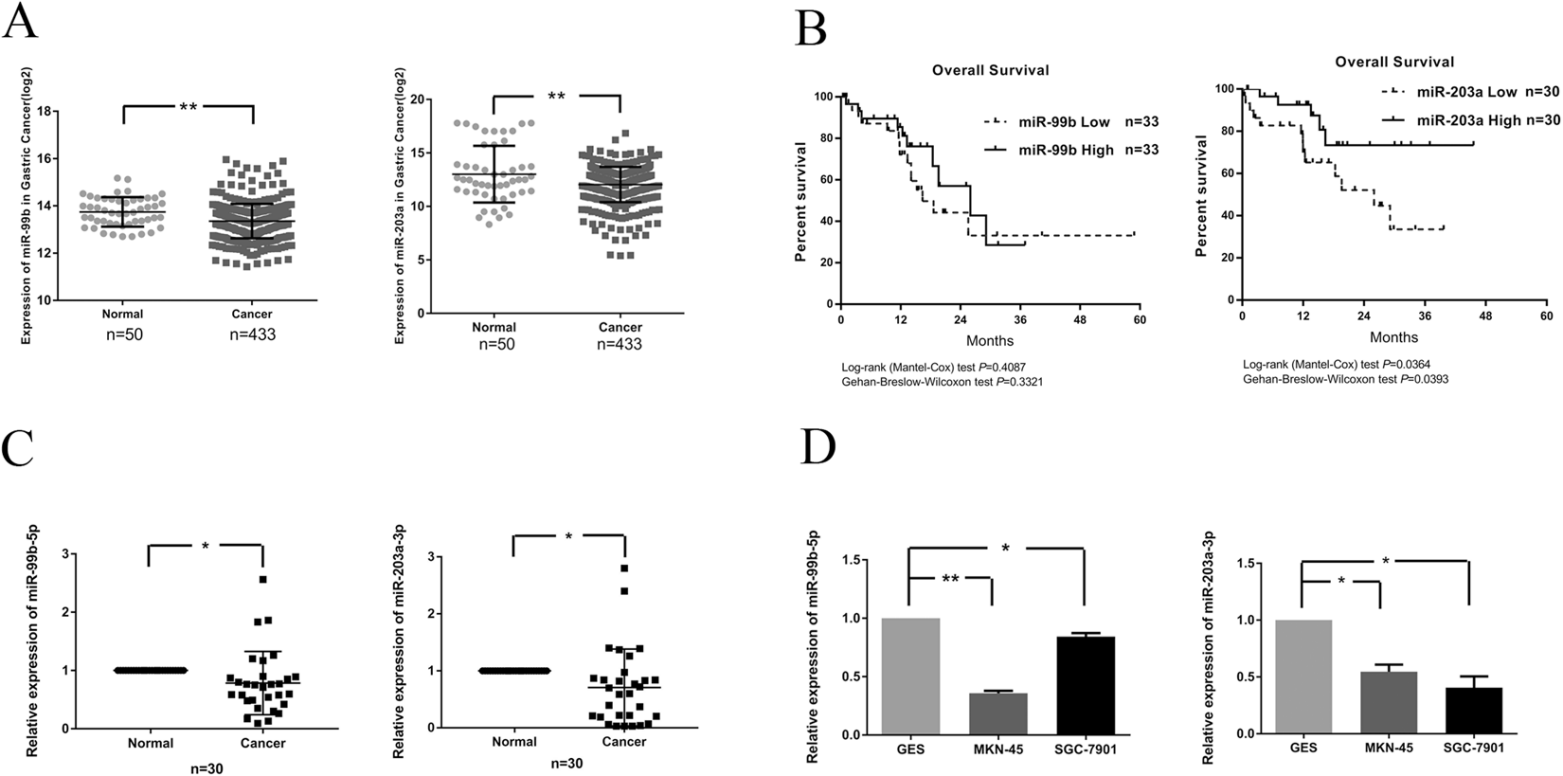
Expression of miR-99b-5p and miR-203a-3p in GC. (**A**) The expression data RNA-Seq analysis of miR-99b and miR-203a expression in GC tissues (n = 50) and normal tissues (n = 433). RNA-Seq analysis used data download from TCGA. (**B**) Overall survival analysis showed that there was no statistically significance between miR-99b high expression and low expression tumors, but miR-203a higher expression tumors had a better prognosis than lower expression tumors (*P* < 0.05). (**C**) Relative expression of miR-99b-5p and miR-203a-3p in GC tissue (n = 30) and matched adjacent normal tissues were determined by quantitative RT-PCR (qRT-PCR). U6 was used as loading control. (**D**) qRT–PCR analysis of miR-99b-5p/203a-3p expression in normal gastric cell (GES) and GC cell lines (MKN-45 and SGC-7901). Each sample was analyzed in triplicate (*P < 0.05, **P < 0.01, Student’s t test).

To determine whether miR-99b-5p and miR-203a-3p were aberrantly expressed in GC, we re-analyzed RNA-seq data that was downloaded from The Cancer Genome Atlas website (TCGA) and found that miR-99b and miR-203a levels were lower in GC tissues than in normal tissue controls (*P* < 0.01) (Fig. 1A). We then performed qRT-PCR analysis in a set of 30 GC tissues and 30 matched adjacent normal gastric tissues. Significantly, a remarkable decrease of miR-99b-5p and miR-203a-3p expression was observed in the GC tissues compared with that in the matching non-tumor tissues. Based on the miR-99b-5p and miR-203a-3p expression levels measured by RT-qPCR, the 30 patients were divided into low (<1) and high (>1) miR-99b-5p/203a-3p expression groups according to the non-tumor tissues using the relative quantification. (Fig. 1C). The correlation between clinicopathologic factors and miR-99b-5p/203a-3p levels was examined in GC samples as shown in Table 1. The data showed that the expression was not associated with gender (*P* = 0.227/0.227), age (*P* = 0.169/ 0.612), lymphatic metastasis histology (*P* = 0.499/0.501), TNM stage (*P* = 0.543/0.743) and histology (*P* = 0.213/0.103), probably owing to the limited number of samples. In addition, as shown in Fig. 1B, the survival time was significantly longer in patients with miR-203a-high expression than that in patients with miR-203a-low expression (*P* < 0.05). However, the survival time did not differ between miR-99b-high and miR-99b-low expression.

**Table 1:** Relationship between miR-99b-5p/203a-3p/IGF-1R expression and clinicopathologic characteristics of GC.

Variables		N	miR-99b-5p		*P* value	miR-203a-3p		*P* value	IGF-1R		*P* value
			High	Low		High	Low		High	Low	
Gender	Male	24	6	18	0.227	6	18	0.227	19	5	0.433
	Female	6	0	6		0	6		4	2	
Age(y)	<50	4	2	2	0.169	1	3	0.612	3	1	0.1
	≥ 50	26	4	22		5	21		20	6	
lymph node metastasis	N0	3	0	3	0.499	1	2	0.501	2	1	0.564
	N1–N3	27	6	21		5	22		21	6	
metastasis	M0	28	5	23	0.366	6	22	0.634	22	6	0.418
	M1	2	1	1		0	2		1	1	
TNM stage	I–II	5	1	4	0.543	3	2	0.743	4	1	0.651
	III	23	4	19		3	20		18	5	
	IV	2	1	1		0	2		1	1	
Tumor Differentiation	Well	1	0	1	0.213	1	0	0.103	0	1	0.195
	Moderate	7	3	4		2	5		6	1	
	Poor	22	3	19		3	19		17	5	

To investigate miR-99b-5p/203a-3p function *in vitro*, we examined their expression in two GC cell lines. As shown in Fig. 1D, SGC-7901 and MKN-45, were characterized with lower expression of miR-99b-5p/203a-3p in mRNA levels compared with the immortalized gastric epithelial cell lines (GES-1) (*P* < 0.05). Together, these data reveal that the downregulation of miR-99b-5p and miR-203a-3p may be involved in GC development.

### Overexpression of miR-99b-5p and miR-203a-3p inhibit proliferation of GC cells

**Figure 2:**
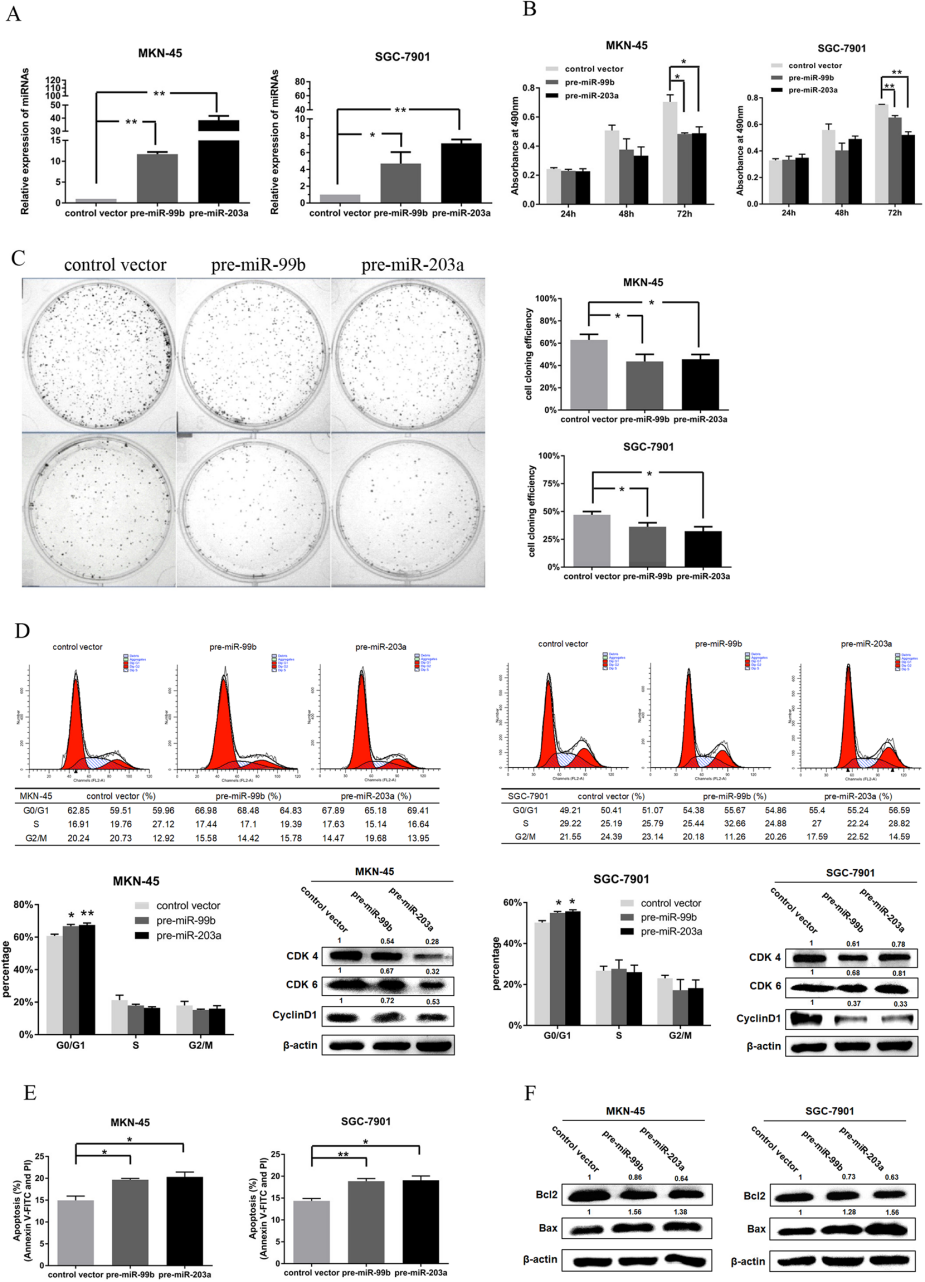
Ectopic expression of miR-99b-5p and miR-203a-3p in GC cells affects cell viability, colony formation, cell cycle and apoptosis *in vitro*. (**A**) Enforced expression of miR-99b-5p and miR-203a-3p was detected by qRT-PCR after transfected with pre-miR-99b and pre-miR-203a vectors in MKN-45/SGC-7901 cells. (**B**) GC cells expressing miR-99b-5p and miR-203a-3p show a decreased viability compared to cells expressing the negative miRNA control. (**C**) The percentage of colonies shows a significantly decrease in cells expressing miR-99b-5p/203a-3p. (**D**) Effects of miR-99b-5p/203a-3p overexpression on the cell cycle progression of MKN-45/ SGC-7901 cells measured by flow cytometric analysis. The expression of Cyclin D1 and CDK4/6 was analyzed by western blot. (**E**) Over-expression of miR-99b-5p/203a-3p significantly promotes cell death in GC cells. (**F**) The expression of Bax and Bcl2 was analyzed by western blot (*P < 0.05, **P < 0.01, Student’s t test or Mann-Whitney test).

To study the biological role of miR-99b-5p/203a-3p in tumor cells, cell growth was evaluated by MTT and colony formation assays. GC cells were transfected with pre-miR-99b or pre-miR-203a plasmid and controls. The transfection efficiency was monitored with GFP-labeled oligo, and an average of 80% efficiency was observed (Supplementary Fig. 1). Successful increase of miR-99b-5p and miR-203a-3p expression in GC cells was confirmed by qRT-PCR (*P* < 0.05) (Fig. 2A).

MTT and colony formation assays showed that significant inhibition of cell proliferation in the miR-99b or miR-203a transfected cells compared with the control vector (*P* < 0.05, Fig. 2B,C). To further address whether the observed changes in proliferation were due to cell cycle and apoptosis, PI staining assay was used. Overexpression of miR-99b-5p/203a-3p repressed cell cycle arrest at G1-S in GC cells (Fig. 2D) and induced cell apoptosis (Fig. 2E).

To further explore the possible molecular mechanisms of miR-99b-5p/203a-3p arrested cell cycle and resulting apoptosis, we detected the expression of cell cycle related regulators and apoptosis-related proteins. After overexpression of miR-99b-5p/203a-3p in GC cells, the results of western blot analysis demonstrated that miR-99b-5p/203a-3p expression reduced the expression of cyclin D1, CDK4/6 (Fig. 2D) and Bcl-2 (Fig. 2F), as well as upregulated Bax. Together, these findings indicate that miR-99b-5p and miR-203a-3p may act as tumor suppressors in GC.

### Inhibition of miR-99b-5p and miR-203a-3p promote proliferation of GC cells

**Figure 3:**
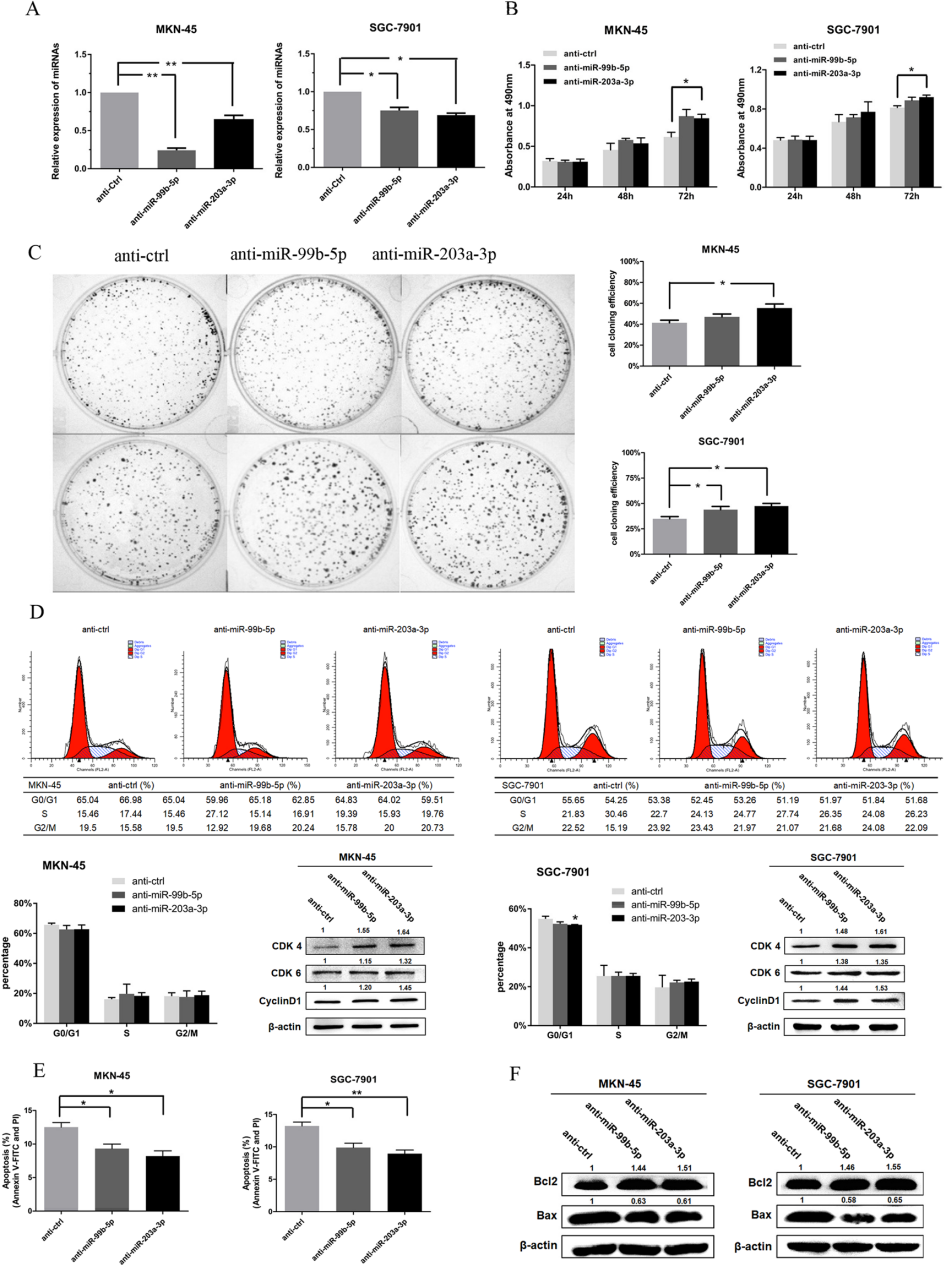
Inhibiting miR-99b-5p/203a-3p contributes to gastric cell growth. (**A**) qRT-PCR analysis of MKN-45/SGC-7901 cells after transfection with anti-miR-99b-5p/203a-3p or a negative control. (**B,C**) MTT assay and colony formation were used to assay the effects of anti-miR-99b-5p/203a-3p in MKN-45/SGC-7901 cells. (**D,E**) Cell cycle and apoptosis were determined in MKN45/SGC-7901 cells transfected with anti-miR-99b-5p/203a-3p or a negative control. The expression of Cyclin D1 and CDK4/6 was analyzed by western blot. (**F**) The expression of Bax and Bcl2 was analyzed by western blot (*P < 0.05, **P < 0.01, Student’s t test).

To investigate the antiproliferative role of miR-99b-5p/203a-3p in SGC-7901 and MKN-45 cells, we eliminated endogenous miR-99b-5p/203a-3p in cells by using their inhibitors. The expression of miR-99b-5p/203a-3p was significantly decreased in GC cells after transfection of their inhibitors (*P* < 0.05, Fig. 3A). Downregulation of miR-99b-5p/203a-3p resulted in slightly increased cell viability, colony formation and a slight difference in cell cycle and apoptosis, which may be due to the low expression of endogenous miR-99b-5p/203a-3p in GC cells (Fig. 3B–E).

In addition, downregulation of miR-99b-5p/203a-3p increased the expression of cyclin D1, CDK4/6 (Fig. 3D) and Bcl-2 (Fig. 3F), as well as decreased Bax expression. These results demonstrate that miR-99b-5p and miR-203a-3p regulate the proliferation, cell cycle and cell apoptosis of GC cells.

### IGF-1R is a co-target of miR-99b-5p and miR-203a-3p

**Figure 4:**
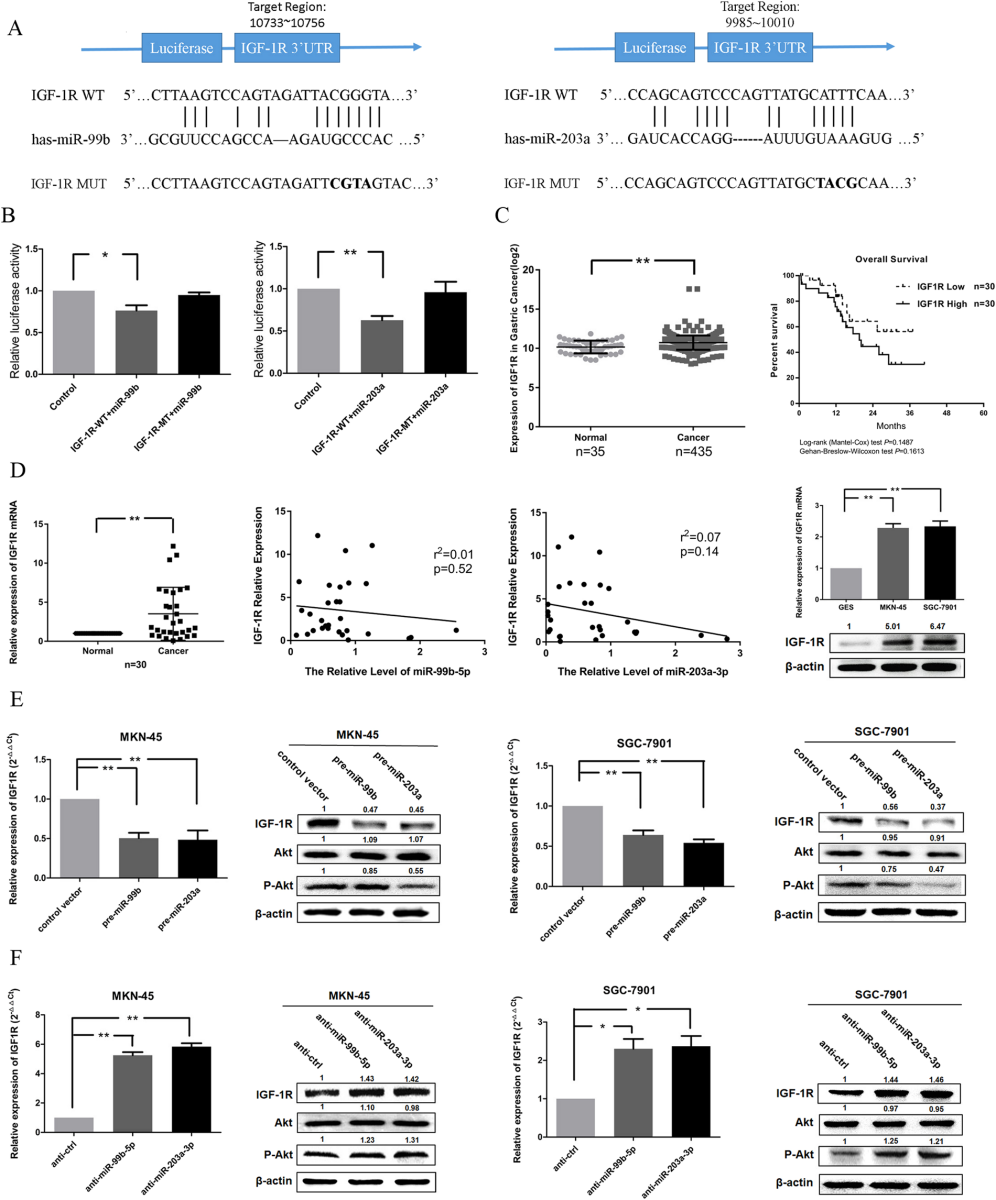
IGF-1R is experimentally validated as a co-target of miR-99b-5p and miR-203a-3p in GC cells. (**A**) Putative miR-99b-5p/203a-3p-binding sites in the IGR-1R 3′UTRs, mutations were generated in the IGF-1R 3′UTR sequences by mutating 4 nt for the seed region of miR-99b-5p/203a-3p, as indicated. (**B**) Dual luciferase assays were performed in HEK293 cells after co-transfection with the wild-type or mutant IGR-1R 3′-UTR plasmids and pre-miR-99b/203a. (**C**) The TCGA data of IGF1R mRNA expression in GC tissues (n = 35) and normal tissues (n = 435). Overall survival analysis showed that there was no statistically significant between IGF1R high expression and low expression tumors. (**D**) IGF-1R was determined by qRT-PCR in GC tissues (left). The correlation between miR-99b-5p/203a-3p and IGR-1R was analyzed. IGF-1R was determined by qRT-PCR and western blot in GC cell lines (right). β-actin was employed as a housekeeping control. (**E,F**) IGF-1R expression level was measured by qRT-PCR and western blot after transfection with pre-miR-99b/203a and anti-miR-99b-5p/203a-3p in MKN-45/SGC-7901 cells (*P < 0.05, **P < 0.01, Student’s t test or Mann-Whitney test).

To further explore the underlying mechanisms by which miR-99b-5p and miR-203a-3p exert their functional effects on GC, we used RegRNA to search for potential downstream targets of these two miRNAs (Supplementary Fig. 2). As shown in Fig. 4A, IGF-1R was selected for some binding sites both of miR-99b-5p and miR-203a-3p, which was observed in the 3′UTR of IGF-1R mRNA. Then, we performed luciferase reporter assay containing wild (WT) and mutated (MUT) IGF-1R 3′UTR. When co-transfected with IGF-1R 3′UTR, miR-99b and miR-203a showed a significant reduction of IGF-1R. In addition, the luciferase activity was unaffected by the miR-99b-mut and miR-203a-mut, indicating that miR-99b-5p and miR-203a-3p may suppress gene expression through their binding sequences at the IGF-1R 3′UTR (Fig. 4B).

Moreover, we found that the IGF-1R level was overexpressed in GC tissues than compared to normal tissue controls from TCGA database (*P* < 0.01). Kaplan-Meier survival curves are shown in Fig. 4C (*P* > 0.05). We then performed qRT-PCR in a set of 30 primary GC tissues and 30 paired non-tumor gastric tissues from the same patients and found that IGF-1R was upregulated in GC tissues. Meanwhile, miR-99b-5p and miR-203a-3p levels were inversely correlated with IGF-1R expression by qRT-PCR assay. Likewise, the same results IGF-1R mRNA and protein overexpressed in MKN-45/SGC-7901 cell lines, in which both miR-99b-5p and miR-203a-3p were downregulated (Fig. 4D). The analysis of the relationship between IGF-1R expression level and clinical features of GC patients is shown in Table 1.

In addition, qRT-PCR and western blot analysis showed that pre-miR-99b-5p/203a-3p markedly reduced, whereas miR-99b-5p/203a-3p inhibitors increased the mRNA and protein expression of IGF-1R and the phosphorylation of AKT (P-AKT) at serine 473. However, there is no difference with the total AKT expression (Fig. 4E,F). Together, these findings strongly suggested that miR-99b-5p/203a-3p regulated IGF-1R-AKT signaling pathway by targeting the IGF-1R 3′UTR in the development of GC.

### IGF-1R is involved in miR-99b-5p/203a-3p regulated cell proliferation in GC cells

**Figure 5:**
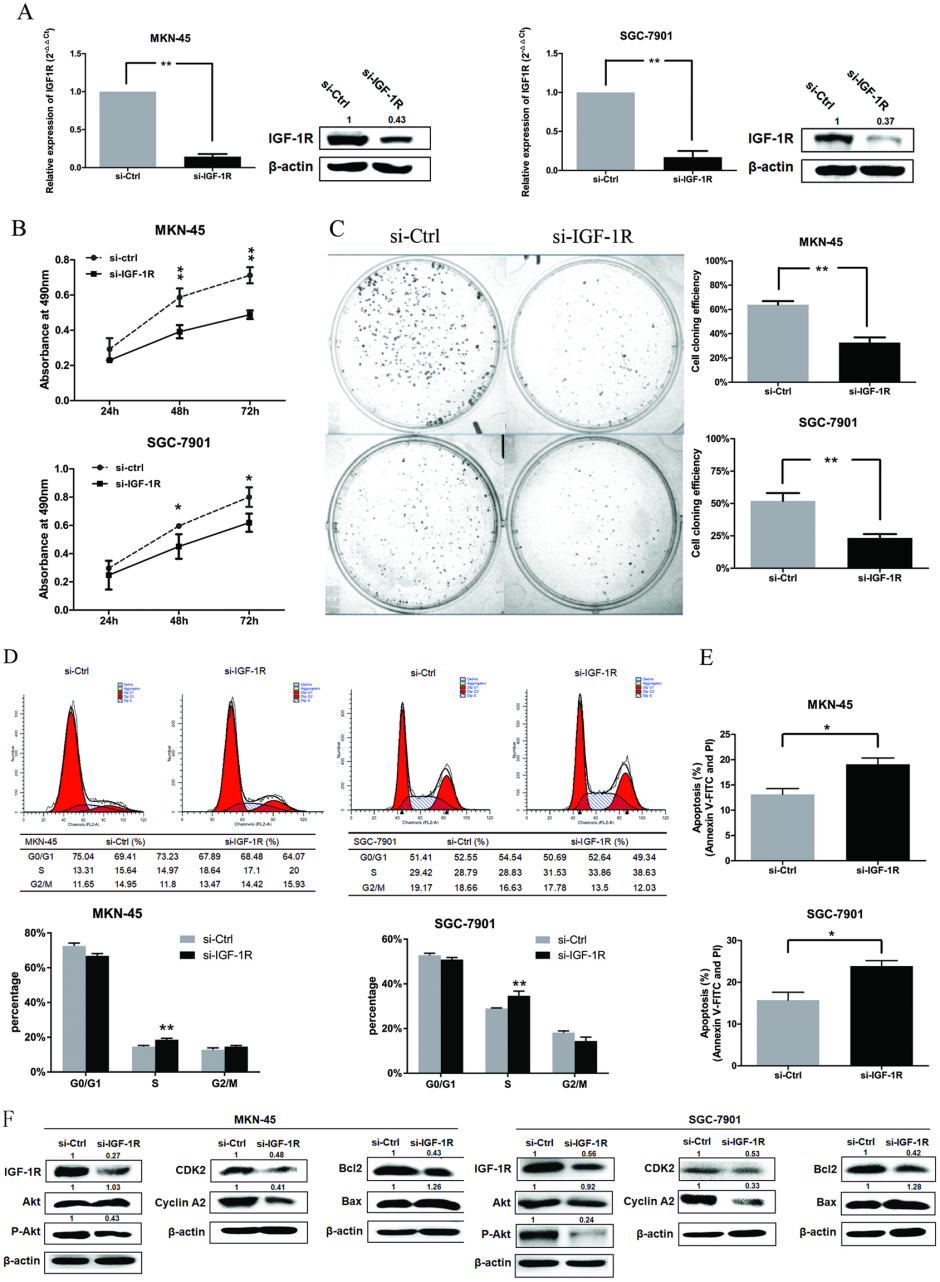
Silencing of IGF-1R could make similar biological function to overexpression of miR-99b-5p/203a-3p in GC cells. (**A**) qRT–PCR (left portion) and western blot (right portion) were performed to determine the expression level of IGF-1R after transfection with IGF-1R siRNA. (**B,C**) MTT assay and clone formation were performed to determine the growth of GC cells treated with si-IGF-1R or a negative control (si-ctrl). (**D,E**) Cell cycle and apoptosis were performed to determine the impact of MKN-45/SGC-7901 cells transfected with si-IGF-1R. (**F**) Expression analysis for IGF-1R/AKT signaling pathway regulation proteins in MKN-45/ SGC-7901 cells at 48 h after transfected with siRNA by western blot (*P < 0.05, **P < 0.01, Student’s t test or Mann-Whitney test).

To evaluate whether IGF-1R was implicated in the antitumor effects of miR-99b-5p/203a-3p in GC, we performed loss-of-function studies by transfection IGF-1R siRNA in MKN-45 and SGC-7901 cells (*P* < 0.05, Fig. 5A). We found that cell proliferation and colony formation (Fig. 5B,C) were significantly decreased after IGF-1R downregulation. Furthermore, silencing of IGF-1R increased the proportion of GC cells in S-phase and induced cell apoptosis (*P* < 0.05, Fig. 5D,E), following the same trend as overexpression of miR-99b-5p/203a-3p. In addition, IGF-1R knockdown markedly inhibited the expression of IGF-1R, P-AKT, Cyclin A2, CDK2, and Bcl2, and it promoted Bax expression (Fig. 5F). These results demonstrated that silencing of IGF-1R and overexpressing of miR-99b-5p/203a-3p shared similar effects in GC cells.

**Figure 6:**
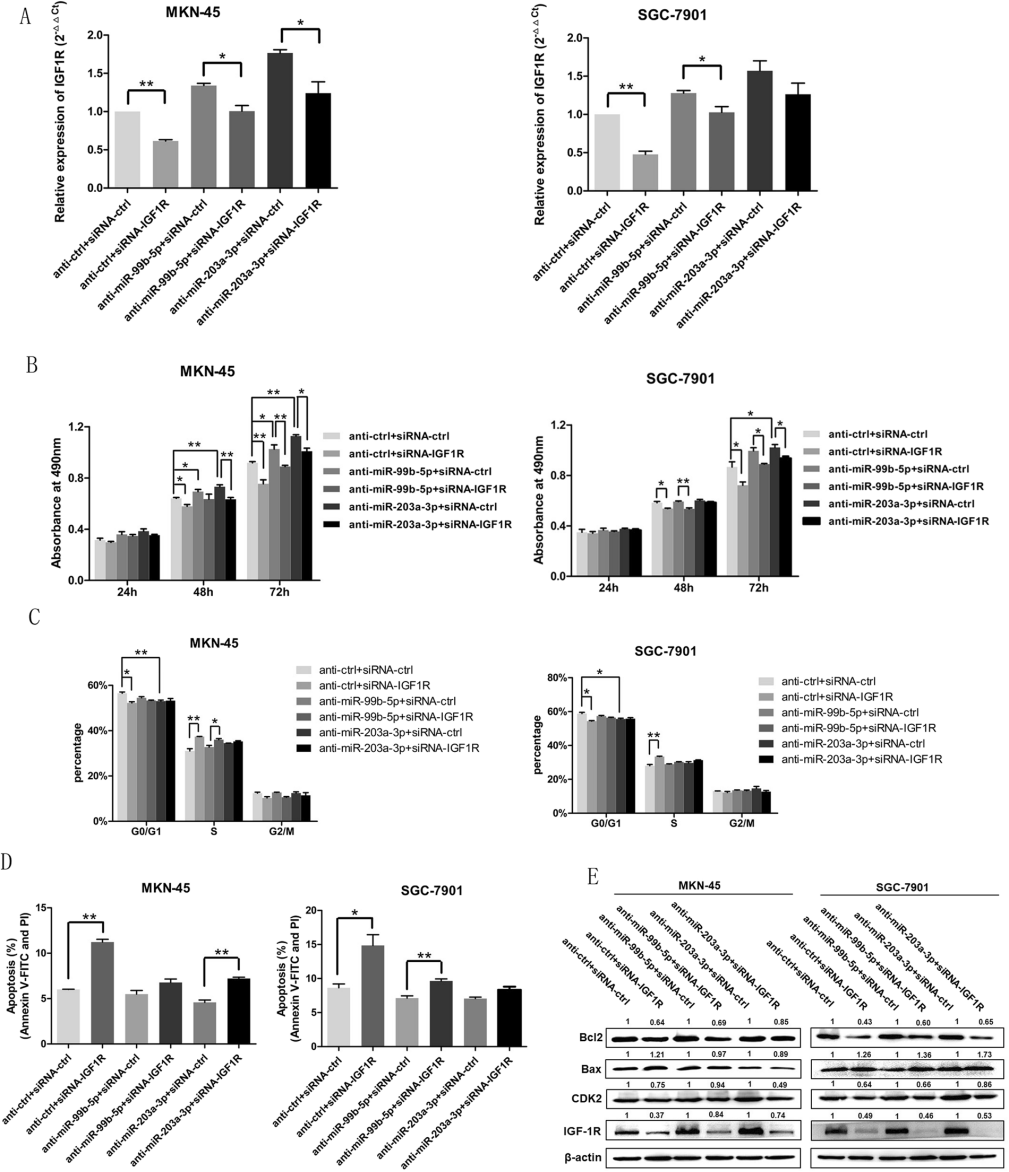
Knockdown of IGF-1R rescues anti-miR-99b-5p/203a-3p–induced cellular phenotypes in GC cells. (**A–E**) qRT-PCR, MTT assay, cell cycle, cell apoptosis and western blot were performed to determine the impact of GC cells treated with anti-miR-99b-5p/203a-3p plus si-IGF-1R expression vectors or related negative controls (*P < 0.05, **P < 0.01, Student’s t test or Mann-Whitney test).

To further explore that miR-99b-5p and miR-203a-3p inhibited tumor progression through targeting IGF-1R, we constructed rescue experiments and co-transfected of si-IGF-1R and anti-miR-99b-5p/203a-3p or their controls in GC cells. Silencing of IGF-1R in GC cells partly rescued the cells from the effects of anti-miR-99b-5p/203a-3p on regulation of IGF-1R expression (Fig. 6A–E). These findings provided additional evidence that miR-99b-5p and miR-203a-3p exhibited tumor suppressor role by directly targeting IGF-1R.

## Discussion

MiRNAs have been regarded as critical regulators in cancer-related processes^29,30^. Increasing evidence have demonstrated that tumor-targeting therapies using miRNAs is becoming a novel diagnostic and therapeutic tool^31^. miR-223, miR-21 and miR-218 have been identified as novel potential biomarkers for gastric cancer detection. In previous studies, miR-99b-5p and miR-203a-3p have been reported to be involved in several cancers^32–34^. For example, miRNA-99b-5p suppresses liver metastasis of colorectal cancer by down-regulating mTOR, and miR-203a suppresses cell metastasis and angiogenesis through VEGFR by targeting HOXD3 in human hepatocellular carcinoma cells^35,36^. Besides, Liu W *et al*. have reported that downregulation of miR-203a by promoter methylation contributes to the invasiveness of gastric cardia adenocarcinoma. However, the underlying mechanisms responsible for decreased expression of miR-99b-5p and miR-203a-3p in GC remain to be determined.

In the present study, we observed that miR-99b-5p and miR-203a-3p were markedly down-expressed in GC tissues and cell lines, which is consistent with the analysis of TCGA. However, the overlap between nontumoral and tumor tissues may be because a large difference in the number of groups. Functionally, based on gain- or loss-of-function assays, our study showed that miR-99b-5p/203a-3p suppressed the cell growth and colony formation, leading to cell cycle arrest and apoptosis in GC cells. Moreover, exogenous miR-203a-3p expression inhibited cell proliferation by decreasing its viability and inhibiting its colony forming capacity more efficiently than miR-99b-5p. In addition, miR-99b-5p/203a-3p altered the expression of the apoptosis/cell cycle-related proteins, including Bcl-2, Bax, CDK4/6, and CyclinD1. Therefore, these results suggest that miR-99b-5p and miR-203a-3p functioned as tumor suppressors for the cell growth in GC. To this end, validation by *in vivo* assays is needed for further research.

To further determine the mechanisms and target mRNAs that were responsible for the suppressive role of miR-99b-5p/203a-3p, we observed that IGF-1R was a target gene of these two miRNAs. Previous studies have showed that IGF-1R is frequently overexpressed in human myeloma^37^, oral squamous cell carcinoma^38^ and breast cancer^39–42^ and that lots of miRNAs coordinate to regulate IGF-1R at multiple levels^43,44^. There were also two reasons for choosing IGF-1R. Firstly, IGF-1R was a co-target gene of miR-99b-5p and miR-203a-3p. Secondly, in our previous study that miR-302b-3p suppresses cell proliferation by targeting IGF-1R in GC^44^.

In this study, using bioinformatic analyses and a luciferase reporter assay, we confirmed that IGF-1R as a direct co-target gene of miR-99b-5p and miR-203a-3p. However, there is no significant inverse correlation between miR-99b-5p/203a-3p and IGF-1R, may be due to the limited number of GC tissue samples. Both mRNA and protein of IGF1R were significantly decreased in miRNAs overexpression group when compared to controls in GC cells. In contrast, we used siRNA to knockdown the expression of IGF-1R and showed that silencing IGF-1R inhibited the cell proliferation and induced cell apoptosis, which is similar to the effect of miR-99b-5p/miR-203a-3p overexpression in GC cells. Interestingly, silencing of IGF-1R in GC cells partly rescued the cells from the effects of anti-miR-99b-5p/203a-3p on regulation of IGF-1R expression and cell proliferation. These results strongly suggested that miR-99b-5p/203a-3p suppress the expression of IGF1R through directly targeting its 3′UTR.

Numerous studies showed that activation of the AKT signaling pathway was essential to the development and progression of cancer. Notably, IGF-1R exerted its function by activating the AKT signaling pathway followed by activation of target genes. In this study, we found that AKT signal pathway was suppressed by miR-99b-5p/203a-3p. Additionally, knockdown of IGF-1R by siRNA could induce similar inhibitory effects with miR-99b-5p/203a-3p on IGF-1R and AKT signal pathway.

In summary, our study showed that aberrant expression of miR-99b-5p/miR-203a-3p could affect cell proliferation of GC cells, probably through IGF-1R and its downstream signal pathway. We hope that our findings for the miR-99b-5p/203a-3p/IGF-1R/AKT signaling pathway will provide valuable information for the development of therapies against GC.

## Materials and Methods

### Gastric tissue samples and cell lines

Human GC tissue samples were obtained from patients undergoing surgical gastric resection at the First Affiliated Hospital of Xi’an Jiaotong University and People’s Hospital of Shannxi Province. The matched non-tumorous tissues were taken from at least 5 cm distance from the edge of tumor tissues. To protocol used in the study was in accordance with the approved guidelines by the ethics committee, Xi’an Jiaotong University, and informed consent was obtained from all individuals. No patients (UICC I-II stages) received preoperative chemo- or radiotherapy before surgery. For UICC III and IV stages, patients agreed to be adjuvant with intraperitoneal chemotherapy to achieve better therapeutic effects. In addition, SGC-7901, MKN-45 and GES-1 cell lines were grown in RPMI-1640 medium (Thermo Scientific HyClone, USA) supplemented with 10% Biological Industries (BI), 10 mg/ml streptomycin (1% P/S) and incubated at 37 °C under a 5% CO_2_ condition.

### RNA extraction and qRT-PCR

Total RNA was prepared from the GC cells and gastric tissues using Trizol reagent (Invitrogen, USA) following the manufacturer’s instruction. The RNA was quantified with a NanoDrop spectrophotometer (USA). Using PrimeScript RT Reagent Kit and SYBR Premix Ex Taq II Kit were purchased from TAKARA (Japan) for the detection of mature miRNAs expression and mRNA expression. The relative expression levels of IGF-1R and miR-99b-5p/203a-3p were respectively normalized to β-actin and U6. PCR was performed by IQ-5^TM^ Real-Time PCR System (Bio-Rad, USA). IGF-1R-specific primers were as follows: forward 5′-TTTCCCACAGCAGTCCACCTC-3′; reverse 5′-AGCATCCTAGCCTTCTCACCC-3′. The relative expression levels were calculated by using the 2^**−**ΔΔCt^ method. All reactions were run in triplicate and all experiments were conducted 3 times.

### Plasmids, siRNA and transfection

For construction of miR-99b-5p (pre-miR-99b), miR-203a-3p (pre-miR-203a) expression vectors and control vector were synthesized with oligo-nucleotides and cloned in between the EcoR I and Hind III sites of the pcDNA6.2^TM^-GW/EmGFP vector (Invitrogen). The miR-99b-5p/203a-3p inhibitors and IGF-1R siRNA were purchased from Gene-Pharma (China). The vector sequences are listed in Supplemental Table 1. For transient transfection, Polyplus reagent (jetPRIME™ Transfection Reagent, Strasbourg, France) was used according to the manufacturer’s protocol.

### Dual-luciferase assay

The 3′UTR of human IGF-1R mRNA was constructed with synthetic oligo-nucleotides and cloned in between the Sac I and Xho I sites of the pmirGLO Dual-luciferase miRNA target expression vector (Promega). HEK293 cells were seeded in 96-well plate and allowed to settle for ~12 h. MiR-99b-5p or miR-203a-3p was co-transfected with the pmirGLO-IGF-1R-3′-UTR-WT or pmirGLO-IGF-1R-3′-UTR-WUT vector into HEK293 cells, respectively. The pmirGLO vector was used as control signals. Then, cells were measured at 24 h after transfection using the Dual-Luciferase Reporter Assay System (Promega, USA).

### Cell proliferation assay

3000 GC transfected cells were transferred to each well of a 96-well plate. At 24 h, 48 h and 72 h after transfection, cell proliferation was measured by the (3–4, 5-dimethylthiazol-2-yl)-2, 5-diphenyltetrazolium bromide (MTT) assay. Cells were stained with 0.5 mg/ml sterile MTT (Sigma–Aldrich, St. Louis, MO, USA) for 4 h and incubated at 37 °C, and then, the medium was discarded, and an extra 150 μl DMSO (Sigma–Aldrich) was added. Cell viability was measured at 24 h, 48 h and 72 h after transfection using FLUOstar OPTIMA (BMG) at 490 nm absorbance.

### Colony formation assay

For the assessment of colony formation, transfected GC cells were seeded in 6-well plates with 1000 cells/well in triplicate and incubated for 1–2 weeks. Then, the plates were washed with PBS and stained with 0.5% crystal violet for 20 minutes. After washing 3 times, the number of colonies was counted by taking pictures from Bio-Rad.

### Cell cycle and cell apoptosis analysis

Cells were cultured in 6-well plates with 2 × 10^5^ cells/well in triplicate and transfected for 48 h, which were then collected and washed with PBS two times and fixed with 70% ethanol at 4 °C overnight. Then, cells were treated with RNase A and propidium iodide (PI) according to KGI Cell Cycle Detection Kit (China). After incubation, the cells were assayed by flow cytometry (Becton, USA). For cell apoptosis analysis, after 48 h transfection, cells were analyzed with Annexin-V FITC Apoptosis Detection Kit (Invitrogen, USA) and examined by using flow cytometer; the data were examined by ModFit software.

### Western blot

Total protein was harvested from GC cells using RIPA buffer (Wolsen, China) after 48 h transfection and 20 μg of isolated protein lysates were separated by 10% SDS-PAGE and transferred to PVDF membrane (Millipore, USA). The membranes were probed with the following primary antibodies: IGF-1R, AKT, phospho-AKT (Ser473), CCND1, CDK4/6, CDK2, Bcl-2 and Bax (Cell Signaling Technology, diluted 1/1,000) overnight at 4 °C. The expression levels of above proteins were standardized to human β-actin using a mouse mAb anti-β-actin antibody. Then, the membranes were incubated with the HRP-conjugated goat anti-mouse or anti-rabbit IgG antibody (ZSGB-BIO, China). The blots were scanned, and the band density was measured using the Quantity One imaging software.

### Statistical analysis

Statistical analysis was performed using the PASW Statistics 17.0 software (SPSS, USA). Each experiment was repeated at least three times. Independent sample Student’s t-test was used if the quantitative data between groups show normal distribution. If not consistent with the normal distribution, using the Wilcoxon-Mann-Whitney test. Pearson’s correlation analysis was used to analyze the correlation between two indices. The relationship between miR-99b-5p/203a-3p/IGF-1R expression level and clinical parameters was calculated by the χ^2^-test. A p-value ≤ 0.05 was considered statistically significant.

## Electronic supplementary material

supplementary information
