# Author Correction: MiR-99b-5p and miR-203a-3p Function as Tumor Suppressors by Targeting IGF-1R in Gastric Cancer

**DOI:** 10.1038/s41598-023-27961-1

**Published:** 2023-01-17

**Authors:** Zhenzhen Wang, Zhenghao Zhao, Yang Yang, Mai Luo, Min Zhang, Xiaofei Wang, Liying Liu, Ni Hou, Qingqing Guo, Tusheng Song, Bo Guo, Chen Huang

**Affiliations:** 1 Department of Cell Biology and Genetics, School of Basic Medical Sciences, Xi’an Jiaotong University Health Science Center, Xi’an, Shaanxi P. R. China; 2 The ART Center, Northwest Women’s and Children’s Hospital, Xi’an, Shaanxi P. R. China; 3 Key Laboratory of Shaanxi Province for Craniofacial Precision Medicine Research, College of Stomatology, Xi’an Jiaotong University, Xi’an, Shaanxi China; 4 Key Laboratory of Environment and Genes Related to Diseases (Xi’an Jiaotong University), Ministry of Education of China, Xi’an, Shaanxi P. R. China

Correction to: *Scientific Reports* 10.1038/s41598-018-27583-y, published online 04 July 2018

This Article contains an error in Figure 4(E), where β-actin should be the same as in Figure 2(F).

The correct Figure 4 and accompanying legend appear below.

**Figure 4:**
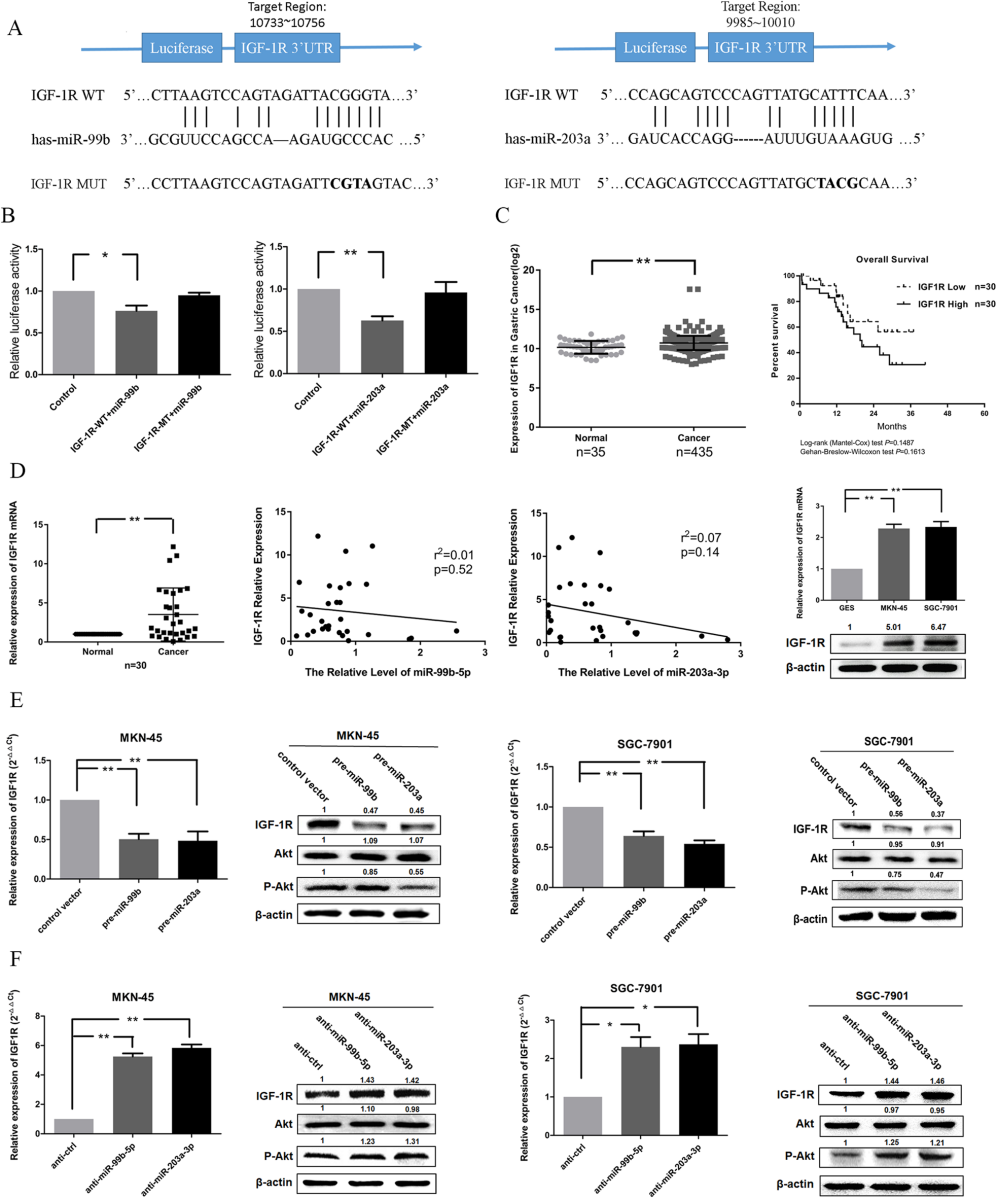
IGF-1R is experimentally validated as a co-target of miR-99b-5p and miR-203a-3p in GC cells. (**A**) Putative miR-99b-5p/203a-3p-binding sites in the IGR-1R 3′UTRs, mutations were generated in the IGF-1R 3′UTR sequences by mutating 4 nt for the seed region of miR-99b-5p/203a-3p, as indicated. (**B**) Dual luciferase assays were performed in HEK293 cells after co-transfection with the wild-type or mutant IGR-1R 3′-UTR plasmids and pre-miR-99b/203a. (**C**) The TCGA data of IGF1R mRNA expression in GC tissues (n = 35) and normal tissues (n = 435). Overall survival analysis showed that there was no statistically significant between IGF1R high expression and low expression tumors. (**D**) IGF-1R was determined by qRT-PCR in GC tissues (left). The correlation between miR-99b-5p/203a-3p and IGR-1R was analyzed. IGF-1R was determined by qRT-PCR and western blot in GC cell lines (right). β-actin was employed as a housekeeping control. (**E**,**F**) IGF-1R expression level was measured by qRT-PCR and western blot after transfection with pre-miR-99b/203a and anti-miR-99b-5p/203a-3p in MKN-45/SGC-7901 cells (*P < 0.05, **P < 0.01, Student’s t test or Mann-Whitney test).

This change does not affect the conclusions of the Article.

