# Supplementary material for: MiR-99b-5p and miR-203a-3p Function as Tumor Suppressors by Targeting IGF-1R in Gastric Cancer
Source: Sci Rep. 2018 Jul 4;8:10119. doi: 10.1038/s41598-018-27583-y (PMC6031697; doi:10.1038/s41598-018-27583-y)
Supplement: Supplementary file 1 — supplementary information [file 41598_2018_27583_MOESM1_ESM.doc]

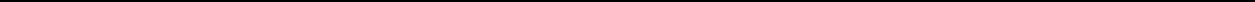


**MiR-99b-5p and miR-203a-3p Function as Tumor Suppressors by Targeting IGF-1R in Gastric Cancer**

^*^Zhenghao Zhao^1, 2^, ^*^Zhenzhen Wang^1, 3^, Yang Yang^1^, Mai Luo^1^, Min Zhang^1^, Xiaofei Wang^1^, Liying Liu^1^, Ni Hou^1^, Qingqing Guo^1^, Tusheng Song^1^, Bo Guo^1^ & Chen Huang^1, 3, 4^

Supplementary data

|  | miRNA | sequence（5’-3’） |
| --- | --- | --- |
|  |  |  |
|  | pre-miR-99b | AATTCGGCACCCACCCGTAGAACCGACCTTGCGGGGCCTTCGCCGCAC |
|  | sense | ACAAGCTCGTGTCTGTGGGTCCGTGTCA |
|  | pre-miR-99b | AGCTTGACACGGACCCACAGACACGAGCTTGTGTGCGGCGAAGGCCC |
|  | antisense | CGCAAGGTCGGTTCTACGGGTGGGTGCCG |
|  |  |  |
|  | pre-miR-203a | AATTCGTGTTGGGGACTCGCGCGCTGGGTCCAGTGGTTCTTAACAGTTC |
|  |  | AACAGTTCTGTAGCGCAATTGTGAAATGTTTAGGACCACTAGACCCGGC |
|  | sense |  |
|  |  | GGGCGCGGCGACAGCGAA |
|  |  |  |
|  | pre-miR-203a | AGCTTTCGCTGTCGCCGCGCCCGCCGGGTCTAGTGGTCCTAAACATTTC |
|  |  | ACAATTGCGCTACAGAACTGTTGAACTGTTAAGAACCACTGGACCCAG |
|  | antisense |  |
|  |  | CGCGCGAGTCCCCAACACG |
|  |  |  |
|  |  | |
|  | Supplemental Table1. The sequences of pre-miR-99b and pre-miR-203a | |
|  | A | B |


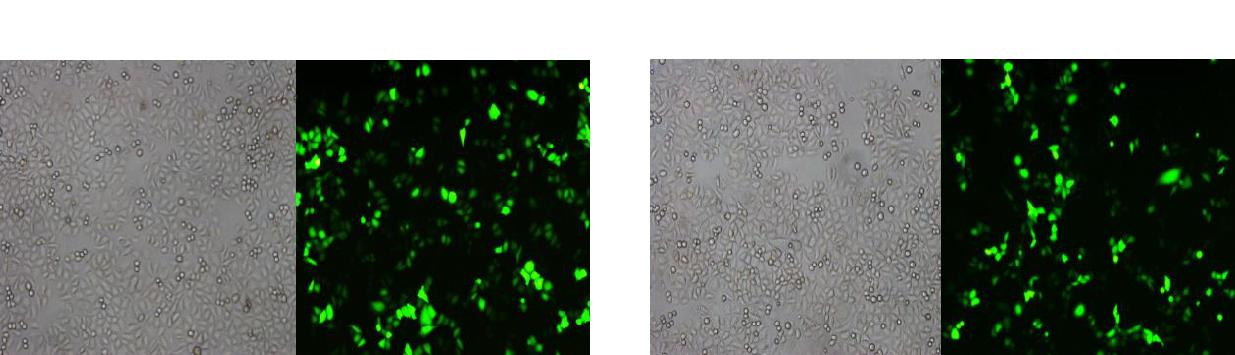


| C | D |
| --- | --- |
|  |  |


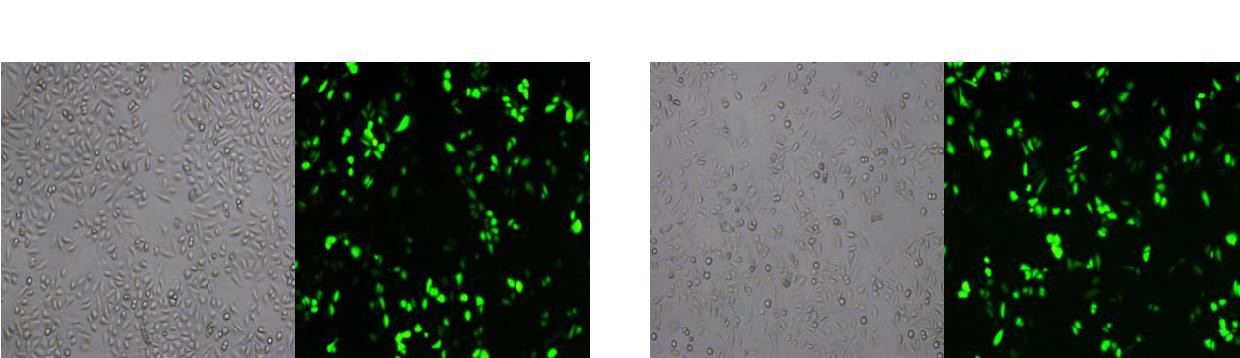


Supplemental Fig.1 The efficiency of transfection with pre-miR-99b and pre-miR-203a (A/B) pre-miR-99b (left) and pre-miR-203a (right) plasmads were efficiencly transfected in MKN-45 cell (A) and SGC-7901 cell (B).


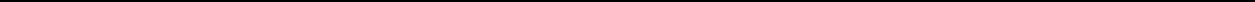

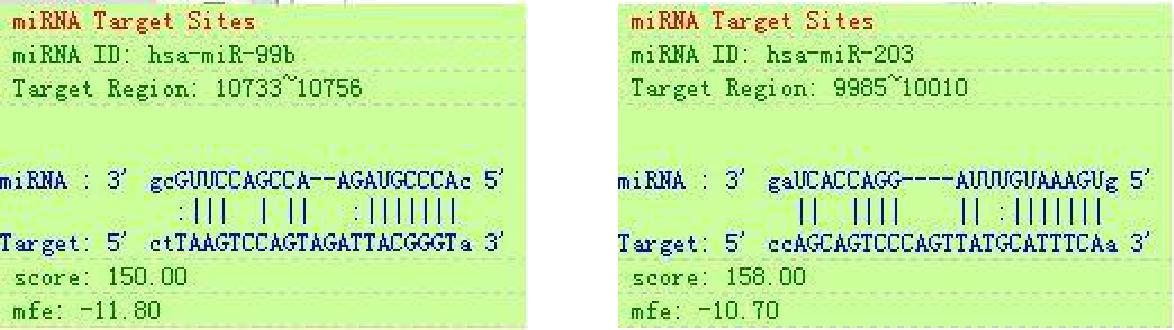


| A | B |
| --- | --- |
|  |  |


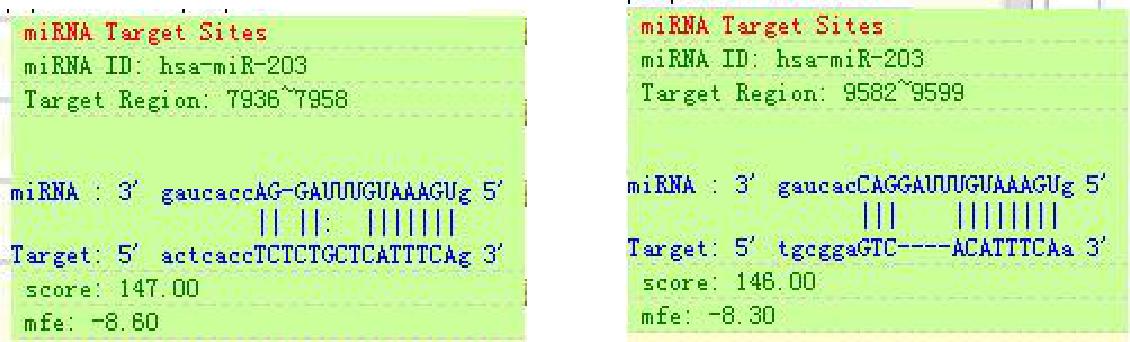


C D

Supplemental Fig.2 IGF-1R was a potential downstream co-target of miR-99b and miR-203a by using RegRNA prediction. (A) miR-99b-5p and IGF-1R target sites. (B-D) miR-203a-3p and IGF-1R target sites.


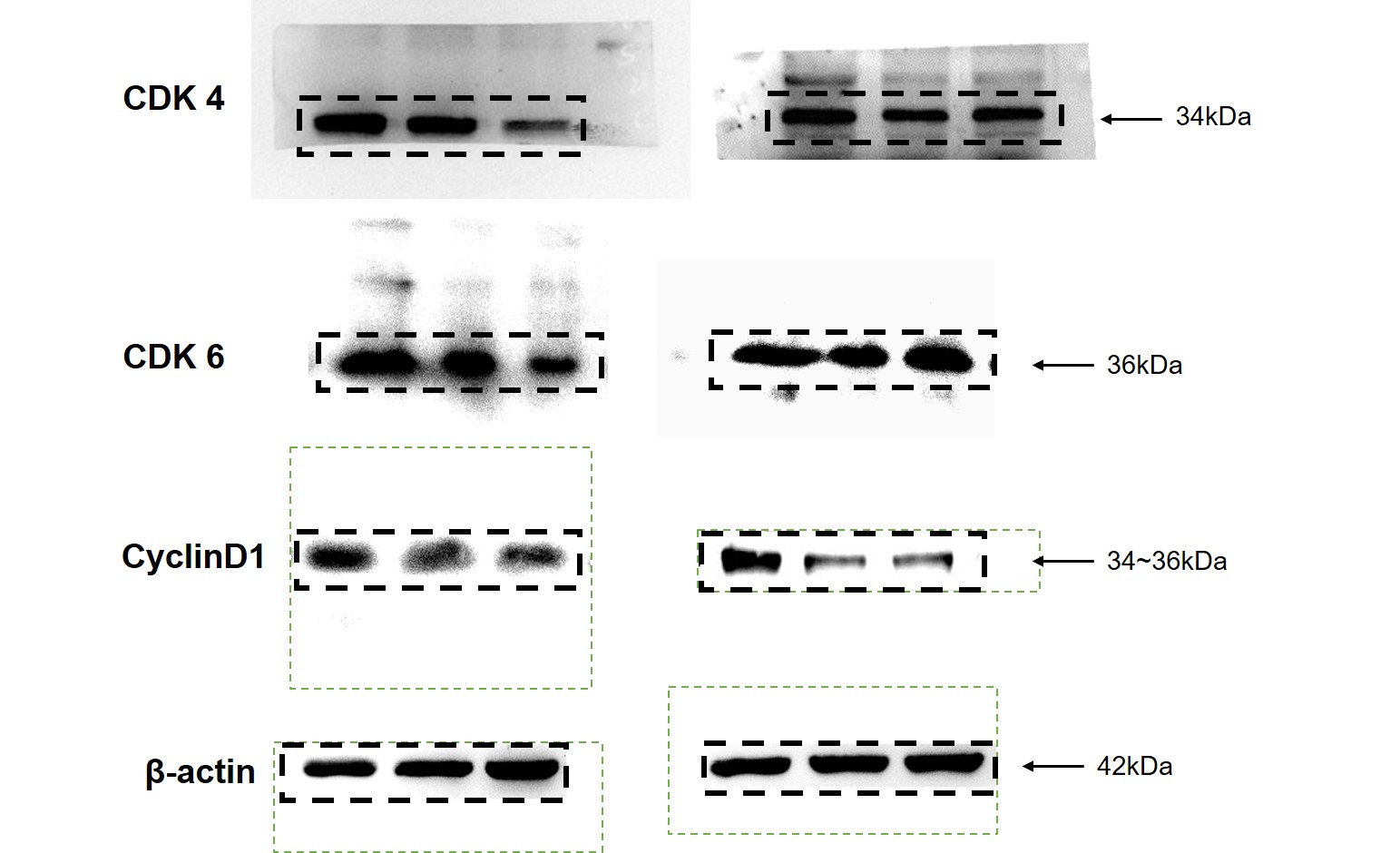


Supplemental Fig. 3 The whole membrane staining for the western blot data in Figure 2D (MKN-45 left, SGC-7901 right), the PVDF membrane was cut according to protein marker after western transfer. Dashed lines indicate how images have been cropped for main figures. Green lines indicate the location of membrane, when it was hard to separate the membrane edges from background.


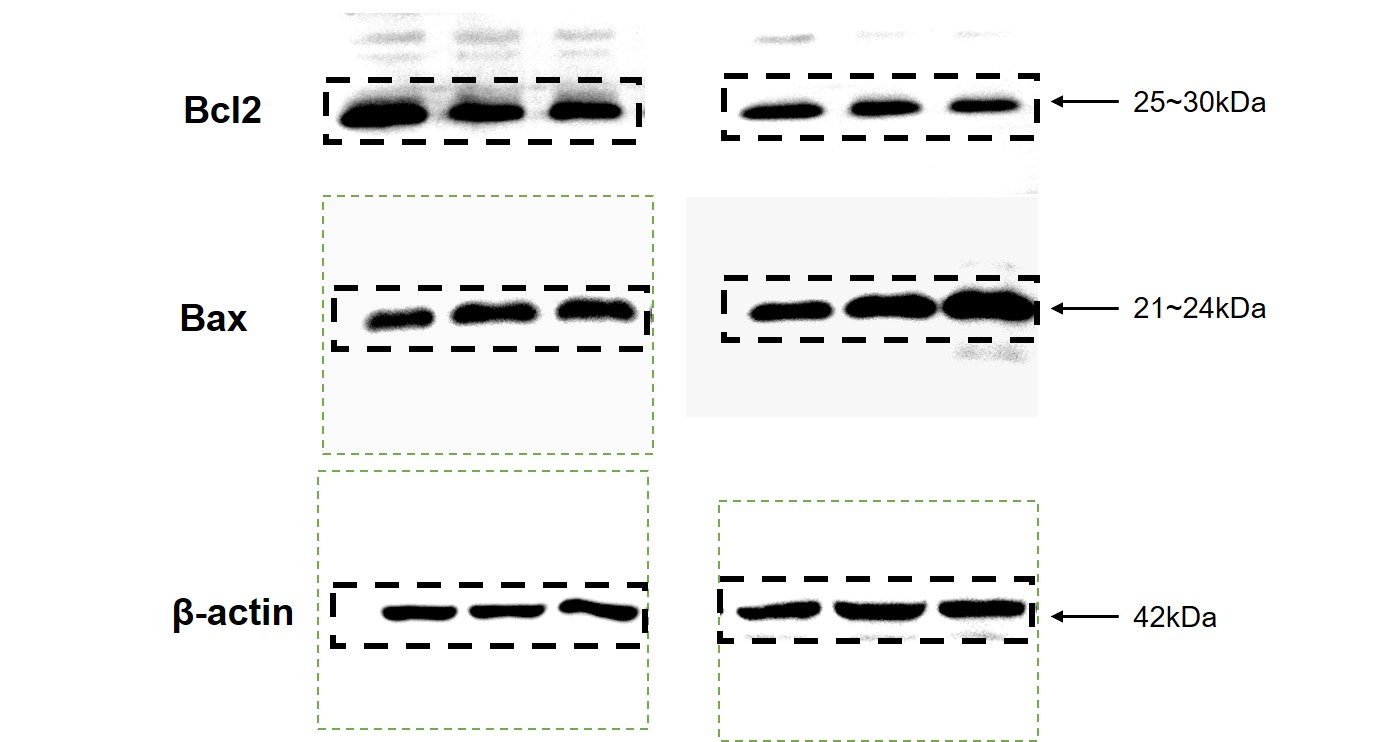


Supplemental Fig. 4 The whole membrane staining for the western blot data in Figure 2F (MKN-45 left, SGC-7901 right), the PVDF membrane was cut according to protein marker after western transfer. Dashed lines indicate how images have been cropped for main figures. Green lines indicate the location of membrane, when it was hard to separate the membrane edges from background.


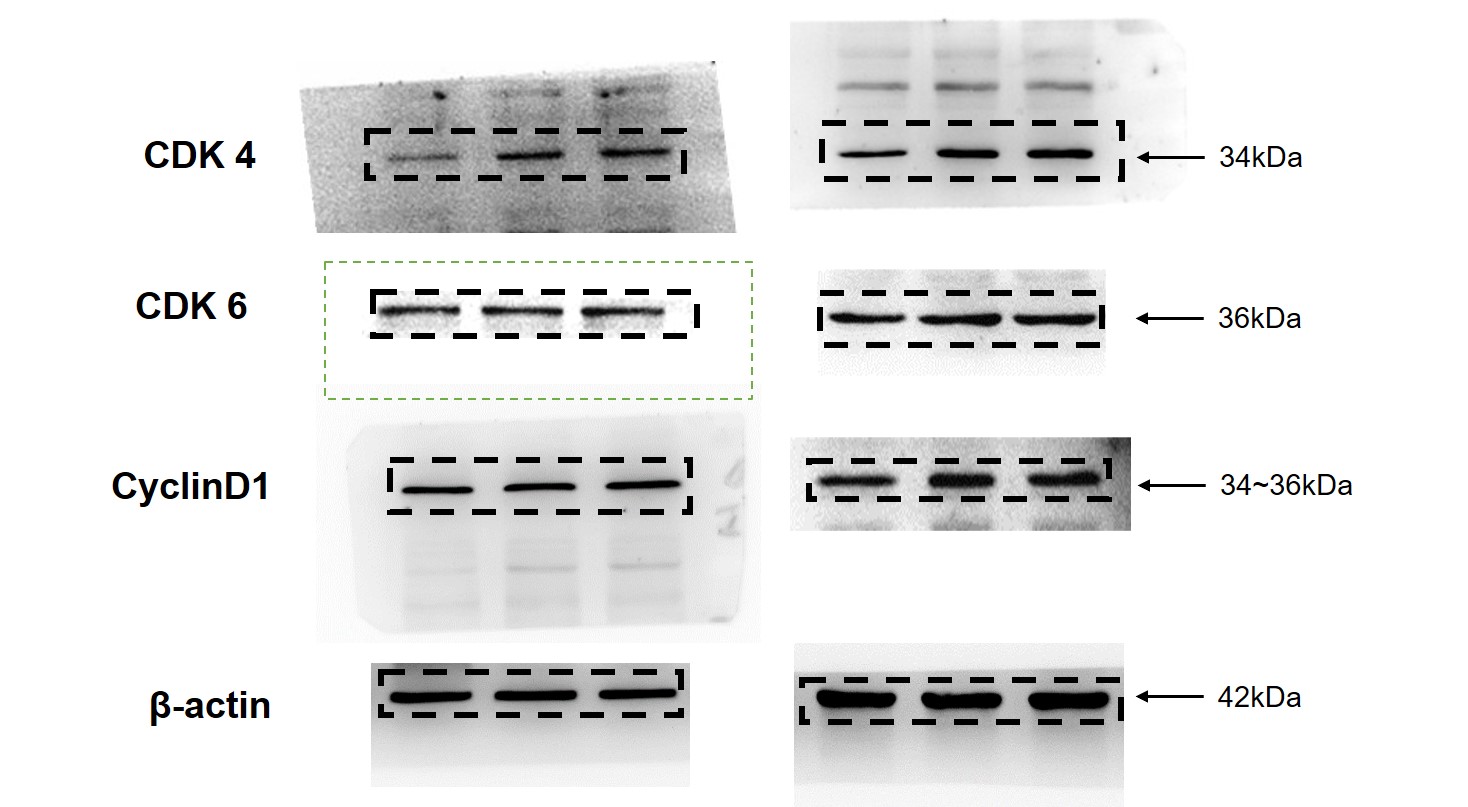


Supplemental Fig. 5 The whole membrane staining for the western blot data in Figure 3D (MKN-45 left, SGC-7901 right), the PVDF membrane was cut according to protein marker after western transfer. Dashed lines indicate how images have been cropped for main figures. Green lines indicate the location of membrane, when it was hard to separate the membrane edges from background.


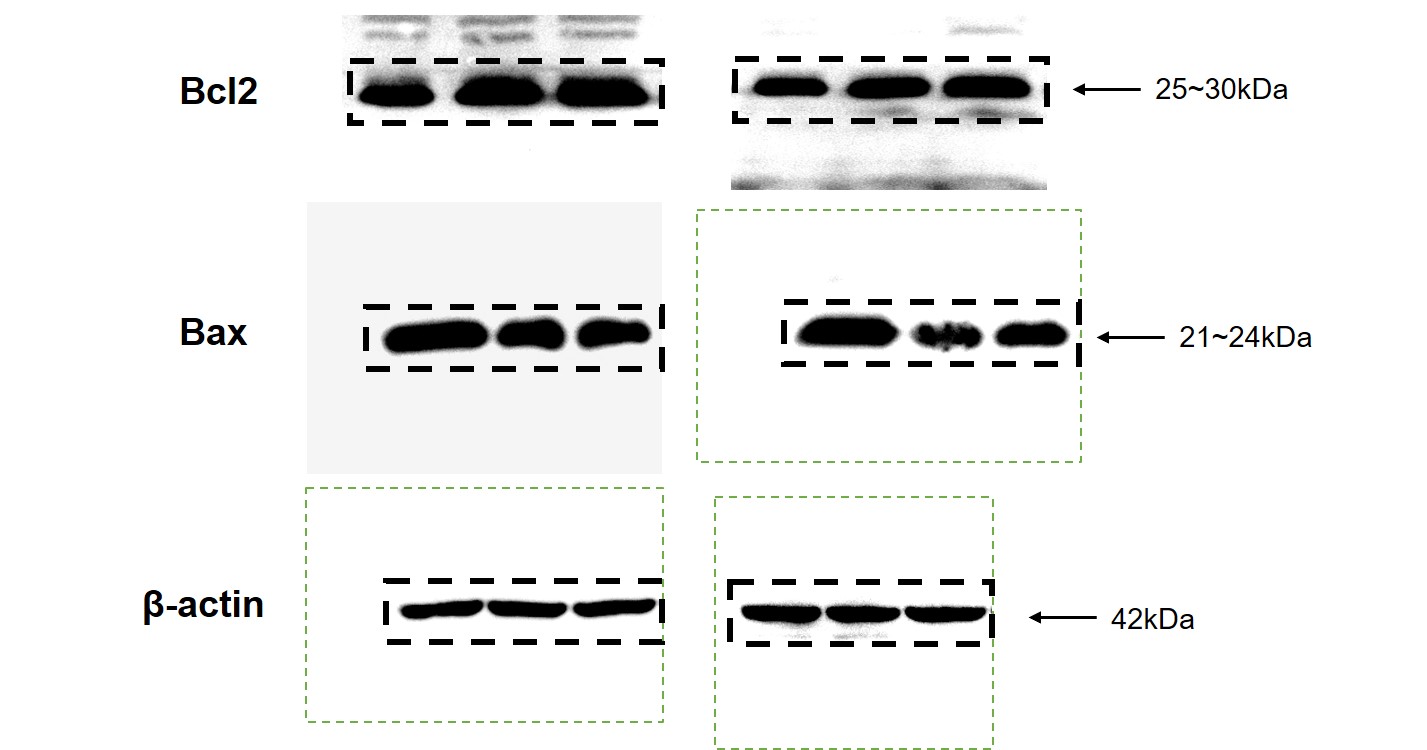


Supplemental Fig. 6 The whole membrane staining for the western blot data in Figure 3F (MKN-45 left, SGC-7901 right), the PVDF membrane was cut according to protein marker after western transfer. Dashed lines indicate how images have been cropped for main figures. Green lines indicate the location of membrane, when it was hard to separate the membrane edges from background.


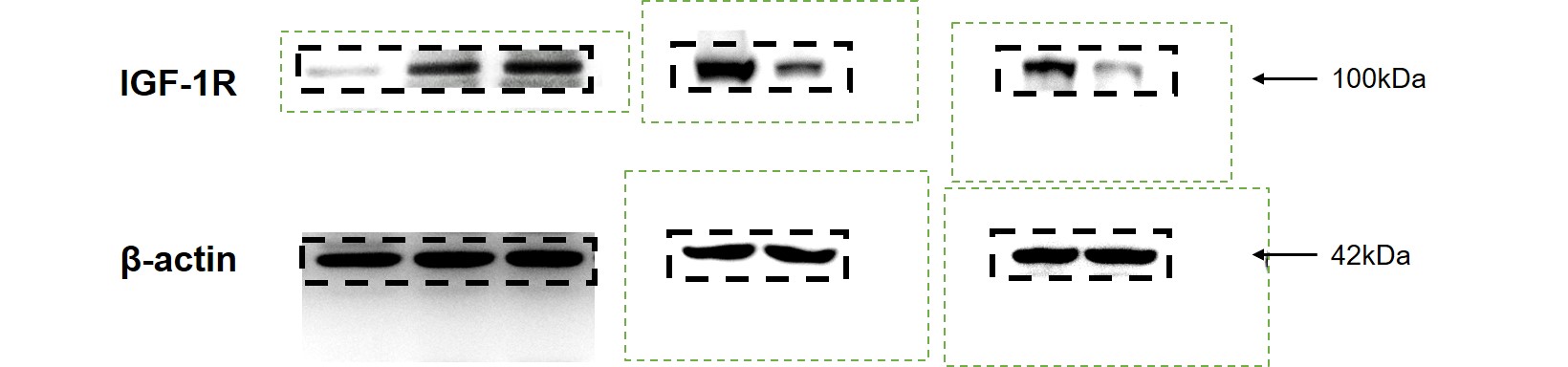


Supplemental Fig. 7 The whole membrane staining for the western blot data in Figure 4D and 5A (MKN-45 left, SGC-7901 right), the PVDF membrane was cut according to protein marker after western transfer. Dashed lines indicate how images have been cropped for main figures. Green lines indicate the location of membrane, when it was hard to separate the membrane edges from background.


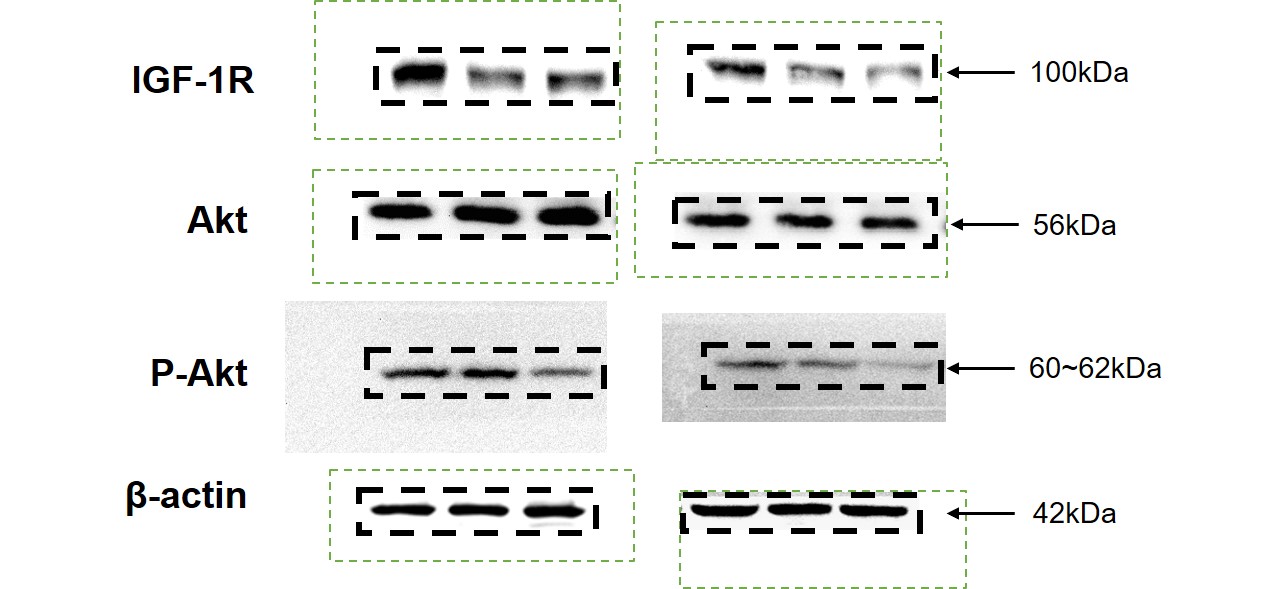


Supplemental Fig. 8 The whole membrane staining for the western blot data in Figure 4E (MKN-45 left, SGC-7901 right), the PVDF membrane was cut according to protein marker after western transfer. Dashed lines indicate how images have been cropped for main figures. Green lines indicate the location of membrane, when it was hard to separate the membrane edges from background.


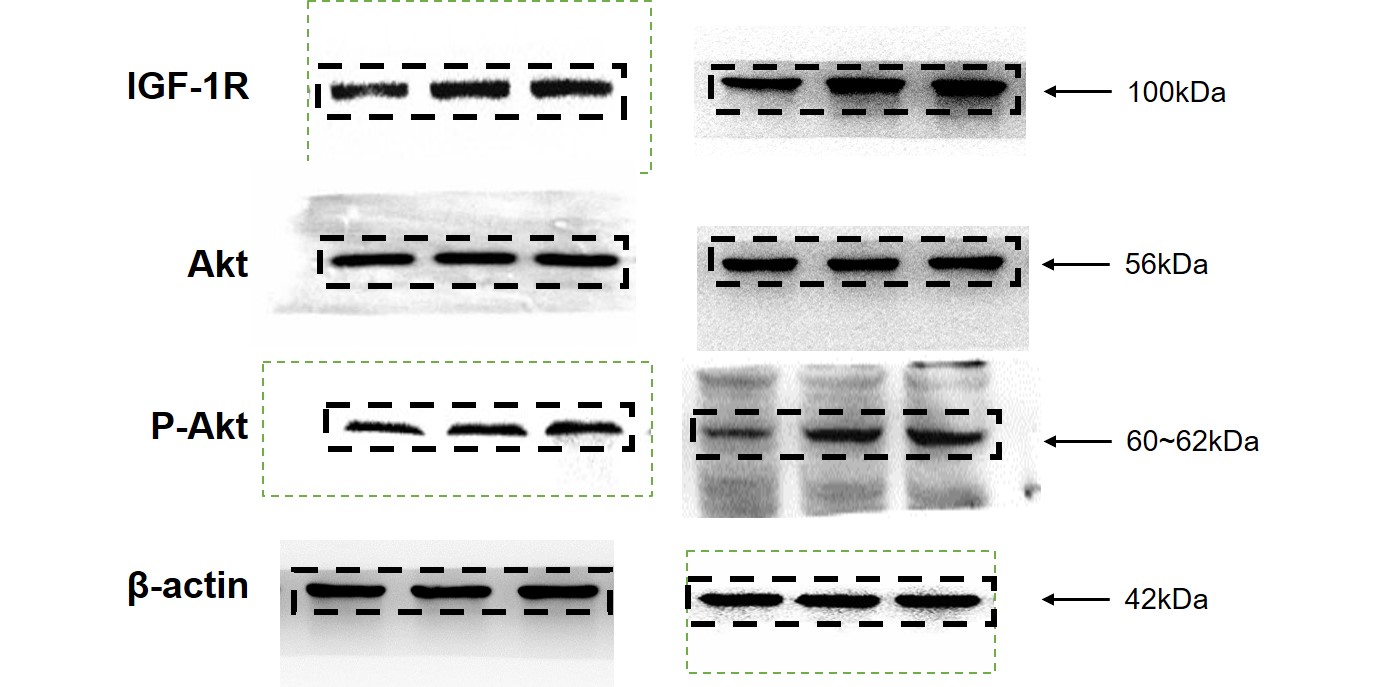


Supplemental Fig. 9 The whole membrane staining for the western blot data in Figure 4F (MKN-45 left, SGC-7901 right), the PVDF membrane was cut according to protein marker after western transfer. Dashed lines indicate how images have been cropped for main figures. Green lines indicate the location of membrane, when it was hard to separate the membrane edges from background.


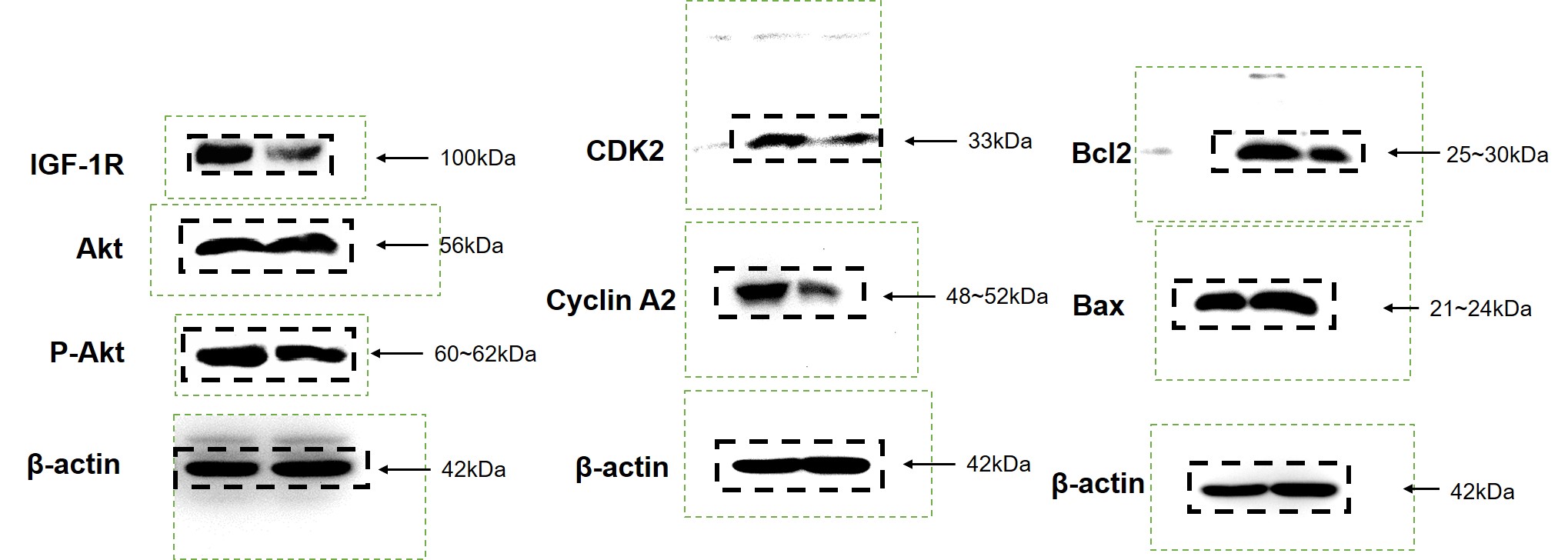


Supplemental Fig. 10 The whole membrane staining for the western blot data in Figure 5F (MKN-45 cell line), the PVDF membrane was cut according to protein marker after western transfer. Dashed lines indicate how images have been cropped for main figures. Green lines indicate the location of membrane, when it was hard to separate the membrane edges from background.


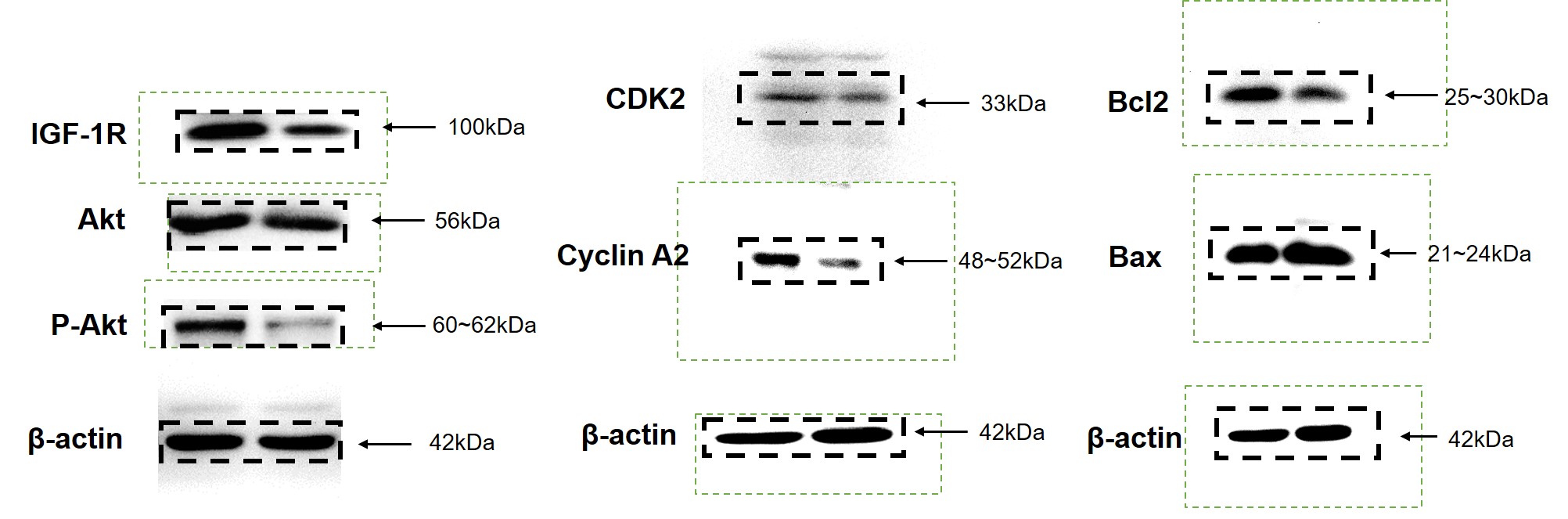


Supplemental Fig. 11 The whole membrane staining for the western blot data in Figure 5F SGC-7901 cell line), the PVDF membrane was cut according to protein marker after western transfer. Dashed lines indicate how images have been cropped for main figures. Green lines indicate the location of membrane, when it was hard to separate the membrane edges from background.


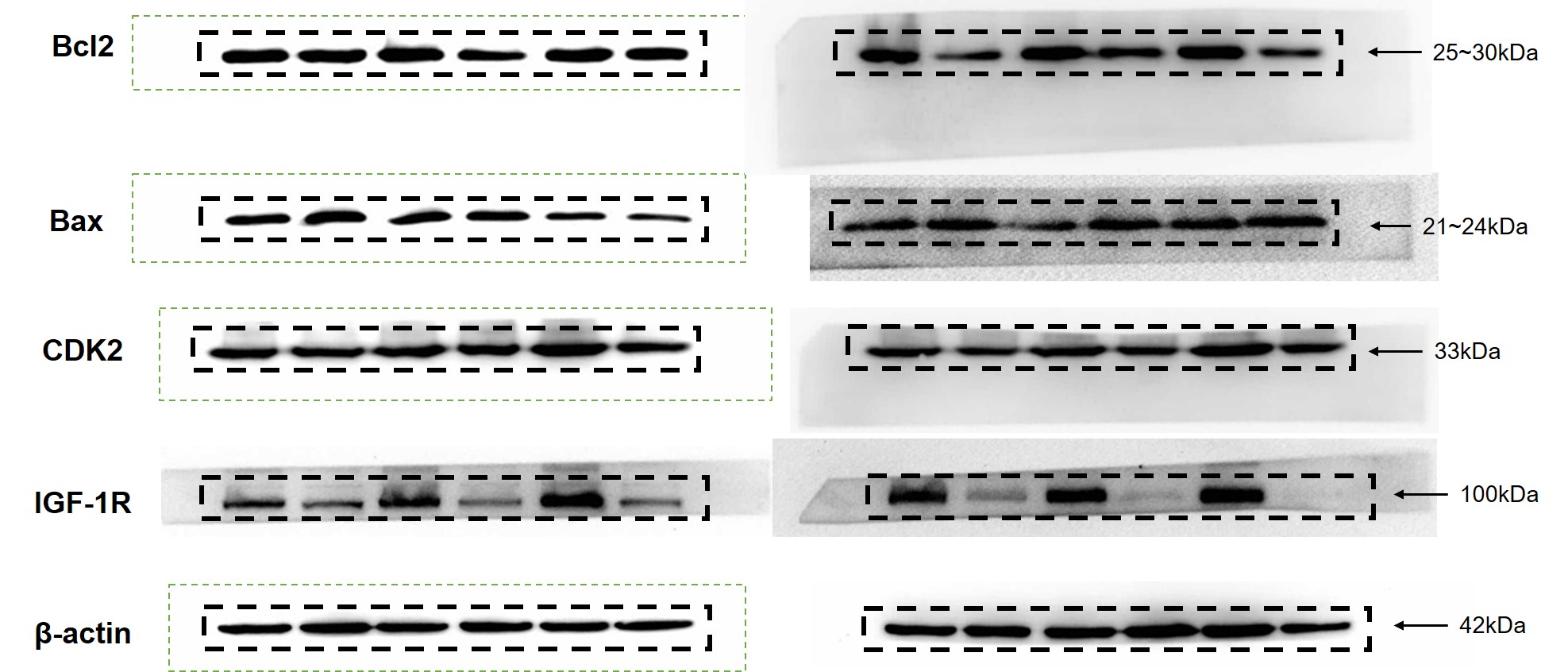
 Supplemental Fig. 12 The whole membrane staining for the western blot data in Figure 6E (MKN-45 left, SGC-7901 right), the PVDF membrane was cut according to protein marker after western transfer. Dashed lines indicate how images have been cropped for main figures. Green lines indicate the location of membrane, when it was hard to separate the membrane edges from background.
